# Effect of a Weight Loss Program on Biochemical and Immunological Profile, Serum Leptin Levels, and Cardiovascular Parameters in Obese Dogs

**DOI:** 10.3389/fvets.2020.00398

**Published:** 2020-08-06

**Authors:** Diego Piantedosi, Anna Teresa Palatucci, Angela Giovazzino, Giuseppina Ruggiero, Valentina Rubino, Nadia Musco, Flavia Carriero, Fortunata Farina, Youssef Abd El Wahab Attia, Giuseppe Terrazzano, Pietro Lombardi, Laura Cortese

**Affiliations:** ^1^Department of Veterinary Medicine and Animal Productions, University of Naples Federico II, Naples, Italy; ^2^Department of Science, University of Basilicata, Potenza, Italy; ^3^Department of Translational Medical Sciences, University of Naples Federico II, Naples, Italy; ^4^Palamara Veterinary Clinic, Naples, Italy; ^5^Department of Agriculture, Faculty of Environmental Sciences, King Abdulaziz University, Jeddah, Saudi Arabia

**Keywords:** canine obesity, leptin, T regulatory cells, left ventricle remodeling, hyperlipidemia

## Abstract

This study aimed to investigate the effects of a weight loss program (WLP) on biochemical and immunological profile, and cardiovascular parameters in a cohort of dogs with naturally occurring obesity. Eleven obese dogs [body condition scoring (BCS), ≥7/9] were enrolled into the study and underwent clinical and cardiovascular examination, and blood testing before (T0) and after 6 months (T1) of WLP. Eleven normal weight (BCS, 4/5) healthy dogs were used as a control (CTR) group. Compared to the CTR group, at T0 obese dogs expressed higher serum leptin concentrations (*p* < 0.0005) that significantly decreased after weight loss (*p* < 0.005) but remained higher than the CTR group. Furthermore, obese dogs showed considerably lower levels (*p* < 0.0005) of regulatory T cell (Treg) compared to the CTR group, but they did not change after weight loss at T1. In obese dogs, tumor necrosis factor (TNF)-α and interleukin (IL)-6 concentrations were substantially reduced at T1 (*p* < 0.0001 and *p* < 0.005). Regarding the cardiovascular parameters, only one obese dog was hypertensive at T0, and systolic blood pressure values showed no significant differences at the end of the WLP. The ratio of interventricular septal thickness in diastole to left ventricle internal diameter in diastole (IVSd/LVIDd) was significantly greater in obese dogs at T0 than in the CTR group (*p* < 0.005). It decreased after weight loss (*p* < 0.05). In obese dogs, troponin I level significantly reduced with weight loss (*p* < 0.05), while endothelin-1 level did not differ statistically. The results suggest that the immune dysregulation in the presence of high leptin levels and reduced number of Treg could affect obese dogs as well as humans. Based on our findings, we may speculate that a more complete immune-regulation restore could be obtained by a greater reduction in fat mass and a longer-term WLP. Finally, left ventricular remodeling may occur in some obese dogs. However, in canine species, further studies are needed to investigate the impact of obesity and related WLP on cardiovascular system.

## Introduction

A common nutritional disease among animals and humans is obesity (1, 2). In industrialized countries, the prevalence of obesity in dogs was reported up to 59% (3, 4) and progressively increased over the years (5). Since the phenomenon of human and canine obesity seems to grow concomitantly in the western world, the adoption of a One Health approach is interesting in order to implement transdisciplinary strategies that could strengthen prevention and control of this disease in both humans and dogs. From this modern and integrated point of view, canine and human obesity does not appear as a disease of an individual but influenced by environmental and socioeconomic factors and deriving from the close interaction between the owner and the dog. A recent survey performed across several European countries has shown that the main common factors associated with obesity in both owners and dogs were represented by increasing age, the lack of physical activity, and unhealthy diet. Furthermore, owners who did not perceive obesity as a pathological condition most likely had an overweight animal (6). Several epidemiological studies that have investigated the risk factors for canine obesity have shown that, in addition to the variables related to the owner (diet choice, feeding method, provision of exercise), also some factors related to the animal (breed, neuter status) are associated with obesity (7). Recently, a higher risk of obesity has been reported in Labrador Retrievers by reason of a documented genetic predisposition (8, 9).

Quality of life is more inferior in human and dogs that are overweight (10), and lifespan can be shortened (11). Obesity can predispose or exacerbate respiratory, orthopedic, metabolic, endocrine, oncological, and cardiovascular disorders in human and dogs (12–17). In veterinary routine practice, canine obesity is a condition difficult to treat and reverse; in addition, successful weight loss is often not maintained. To achieve the goals of a weight loss program (WLP), an appropriate communication and cooperation between veterinarians and owners is needed, and veterinarians must make owners understand that obesity is a serious disease and, if it is not properly treated, can have dangerous consequences for their dog. In addition to being subjected to a dietary treatment, obese dogs must undergo a clinical screening to highlight specific pathological conditions related to the overweight status that require a specific therapy (e.g., orthopedic disorders).

Often, the biochemical profile of obese dogs is characterized by dyslipidemia, with an increased risk of onset of pancreatitis or gastrointestinal disorders over time (18–20). Furthermore, in a subset of obese dogs, the overweight condition can induce the obesity-related metabolic dysfunction (ORMD) (21), similar to the metabolic syndrome (MetS) arising in human beings (22), and it is still under discussion whether this dysmetabolic status in dogs may represent a set of risk factors as in humans.

Regarding cardiovascular disorders, recent studies reported increased systolic blood pressure and left ventricular concentric remodeling in obese dogs (19, 20, 23), similarly to what is well-known to happen in humans (24). In obese dogs, the effects of weight loss on cardiac structure and function are still unclear. In an experimental model of canine obesity, Pelosi et al. (25) reported a reduction in the heart rate and reversibility of concentric hypertrophy associated with caloric restriction and fat loss, and such effects were more evident in animals undergoing exercise. Broussard et al. (18) reported experimental evidence of impaired cardiac function in dogs fed with a diet rich in saturated and monounsaturated fatty acids.

Leptin is a hormone secreted by white adipose tissue that mainly acts at hypothalamic level reducing feed intake and increasing energy metabolism (26). The increased leptin blood levels are strictly related to the metabolic dysfunction and pathological conditions observed during obesity, and in humans, hyperleptinemia can be considered a feature of MetS (17). Leptin has also multiple effects on neuroendocrine function, hematopoiesis, and immune response (27–29). In particular, it regulates the adaptive immunity, influencing activities of T helper (Th) 1 and 2 lymphocytes (30, 31). The hormone stimulates the Th1 production of proinflammatory cytokines, such as interleukin (IL)-2, interferon (IFN)-γ, tumor necrosis factor (TNF)-α, and IL-6 and inhibits the regulatory CD3^+^CD4^+^CD25^+^ Foxp3^+^ T cell population (Treg) (32–34). Treg cells inversely correlate with serum leptin (35) and are significantly reduced in serum samples of obese humans (36). Palatucci et al. (37) described the inverse relationship between the concentration of leptin in serum and circulating Treg levels in obese Labrador Retrievers, suggesting a high similarity with human obesity (35).

In human beings, the development of pathological conditions with high morbidity and mortality (for example, atherosclerosis) are closely related to the persistent slow-grade inflammation and immune dysregulation that occurs in obesity. Altered levels of leptin, adiponectin, cytokines, and acute-phase proteins are well-known in obese humans (38). These pathophysiological evidence have been described also in domestic animals and mouse (39–41). In dogs, several reports indicated that the level of leptin in serum correlates with obesity scale and decreases with weight loss (42–44). Meanwhile, the behavior of other inflammatory markers in canine obesity during weight loss is still unclear (45–48), as well as the relationship between obesity, leptin, circulating Treg levels, systemic inflammation, and immune profile in naturally obese dogs undergoing a weight loss program (WLP) remains poorly understood.

This study investigated the effects of WLP on biochemical and immunological profile, blood leptin level, and cardiovascular parameters in a cohort of dogs with naturally occurring obesity, in order to address the effects due to the imbalance of these aspects on the clinical presentation and management of canine obesity.

## Materials and Methods

### Animals and Study Design

Eleven obese, but otherwise healthy dogs, 10 females (8 spayed) and 1 male (neutered), as well as 11 healthy dogs at ideal body condition, 7 females (3 spayed) and 4 males (1 neutered), were enrolled into this study. The animals were from a client-owned referral population of the Veterinary Teaching Hospital, Department of Veterinary Medicine and Animal Productions (University of Naples Federico II), from November 2018 to November 2019. Written informed consent was obtained from the owners before the beginning of the trial, including a commitment to maintain the required management conditions during the study.

Each recruited dog was classified according to a body condition scoring (BCS) assessed by the same operator (LC), utilizing a 9-point scale system (49). Animals with a BCS ≥7 were considered obese (21), forming the obese group, while animals with a BCS 4/5 were regarded as a healthy weight and included in the control group (CTR group). Based on the guidelines suggested by Tvarijonaviciute et al. (21), dogs were considered to be affected by ORMD when BCS was ≥7/9 and at least two of the following parameters were present: triglycerides, >200 mg/dl; total cholesterol, >300 mg/dl; glucose, >100 mg/dl; and systolic arterial blood pressure, >160 mmHg.

Both groups were homogeneous by age (dogs younger than 2 years or older than 10 years were ruled out). Mean age and weight at the enrollment were respectively 5 ± 2.4 years and 28.3 ± 15.8 kg for the obese group, while they were respectively 5 ± 1.8 years and 22.3 ± 16.2 kg for the CTR group. Regarding dog breeds, the obese group included six mixed breed, three Labrador Retriever, one Jack Russell, and one Epagneul Breton, while the CTR group included four mixed breed, two Golden Retriever, one Jack Russell, one Corso dog, one Labrador Retriever, one Bullmastiff, and one Maltese. All the recruited dogs were considered clinically healthy, based on the history and physical examination, including measurement of arterial blood pressure and complete cardiovascular evaluation. Exclusion criteria were endocrine, liver, renal, heart, and infectious diseases or inflammatory conditions. Animals that were in some physiological conditions, such as pregnancy or nursing, and those under pharmacological treatment and following a regular exercise program were ruled out.

Body condition score, bodyweight evaluation, morphometric measurements (50), complete blood count (CBC), serum biochemical panel, and urinalysis were determined. Besides, the cardiovascular biomarkers endothelin-1 (ET-1) and troponin I (cTnI), electrocardiography (ECG), echocardiography, and arterial blood pressure (ABP) were recorded. Furthermore, insulin, leptin, cytokines (TNF-α and IL-6), and blood immunophenotype (CD3^+^CD4^+^, CD3^+^CD8^+^ T cells, CD4/CD8 ratio, CD21^+^ B cells, Treg cells) evaluations were acquired.

### Weight Loss Program

Obese dogs underwent 6 months WLP using a prescription dry dog feed diet for weight management. Diet was high in protein but low in fat and carbohydrates; therefore, it encouraged steady and effective fat loss while maintaining a patient's muscle mass, promoting satiety, and stimulating resting metabolic rate. Besides, the diet was high in complex carbohydrates and fibers to help even out blood sugar levels throughout the day. The composition of this diet is shown in Table 1. Daily feeding quantities were determined thanks to the manufacturer's label feeding recommendations for adult weight loss. Current enrolled obese dog's diets were gradually switched to a therapeutic weight-loss diet over 7 days to avoid gastrointestinal disturbances. At the time of enrollment, the diet of all the CTR dogs was a high-quality commercial adult maintenance dry food. The total amount of daily food was divided into two meals for the dogs of both groups. The owners were instructed to present the meal to their dogs at 10:00 a.m. and at 7:00 p.m. The dogs were brought to visit at the beginning of the trial and after 6 months; during this time, the owners were contacted monthly by phone, and the feed amount was adjusted according to the weight changes they reported.

**Table 1:** Chemical composition of the experimental diet administered to obese group.

**Parameter**	**Unit**	**As fed**	**g/100 kcal ME**
Moisture	%	7.5	–
Protein	%	29	9.8
Fat	%	6.05	2.0
Linoleic acid	%	1.4	0.5
ω-3	%	0.09	0.03
DHA	%	0.01	0.0
Carbohydrates	%	41.0	13.8
Starch	%	23.1	7.8
Total sugars	%	1.7	0.6
Crude fiber	%	10.0	3.4
Soluble fiber	%	1.6	0.5
Insoluble fiber	%	18.7	6.3
Vitamin A	IU/kg	21,936	741
Vitamin D3	IU/kg	962	32.5
Vitamin E	IU/kg	300 IU/kg	10.1
^a^Metabolizable energy (ME)	kcal/g	2.96 kcal/g	–

a *Calculated following NRC 2006 equations*.

*Ingredients: maize, soya meal, dehydrated poultry protein, barley, wheat gluten meal, pea fiber, cellulose, digest, minerals, animal fat*.

### Body Composition Analysis and Morphometric Measurements

Bodyweight evaluation and morphometric measurements (50) were recorded for obese dogs group before (T0) and after 6 months (T1) of the WLP.

Morphometric measurements were obtained to apply the equations developed to predict lean body mass (LBM) (kg), fat mass (FM) (kg), and body fat percentage (BFP) according to Witzel et al. (50). A tailor's flat tape was used, and all the measurements were taken in centimeters by the same investigator (LC). Forelimb length was measured from the proximal aspect of the metacarpal pad to the point of the elbow. In addition, the hind limb length was estimated from the proximal aspect of the metatarsal pad to the dorsal tip of the calcaneal tuber. Moreover, the head circumference was taken at the widest part of the head between the eyes and ears. Thoracic circumference was determined behind the elbows, in the most convex part. Pelvic circumference was measured in the approximate area of the fifth lumbar vertebra (50).

### Blood Sample Collection

Complete blood count, serum biochemical panel, cardiovascular biomarkers (ET-1 and cTnI), insulin, leptin, cytokines (TNF-α and IL-6), and blood immunophenotype (CD3^+^CD4^+^, CD3^+^CD8^+^ T cells, CD4/CD8 ratio, CD21^+^ B cells, Treg cells) evaluations were acquired in the obese group before (T0) and after 6 months of the WLP (T1). In the CTR group, the above determinations, except cytokines, were performed at the enrollment (T0).

Blood sample collection was cruelty-free, according to the national legislation. Ethical committee approval was required (see ethics statement). Ten milliliters of blood was collected by jugular venepuncture after 12 h of fasting at 9:00 a.m. for both obese and CTR groups. Blood sample obtained was divided immediately into three aliquots. The first aliquot was sited in tubes containing potassium ethylenediaminetetraacetic acid (EDTA) for CBC, performed within 30 min from the collection. The second aliquot of blood was collected similarly in anticoagulated tubes containing EDTA and stored at room temperature up to 5–6 h before immunological assays. The third aliquot was retained in tubes without anticoagulant, allowed to clot, and centrifuged at 908 g for 15 min at 4°C to obtain blood serum. Serum samples were stored at −80°C and defrosted immediately before biochemical profile, cardiovascular biomarkers, insulin, leptin, and cytokines evaluation.

### Complete Blood Count and Serum Biochemistry

The CBC was performed using a semiautomatic cell counter (Genius S, SEAC Radom Group). A semiautomatic chemical chemistry analyzer (OLOT, Spinreact) was utilized to measure blood urea, creatinine, glucose, triglycerides (TG), total cholesterol (T-Chol), alanine aminotransferase (ALT), alkaline phosphate (ALP), total bilirubin (T-Bil), sodium, potassium, albumins, and total proteins (TP), using reagents from Spinreact (Girona, Spain). Serum protein electrophoresis was quantified using a densitometer analyzer (Selvet 24, SELEO S.r.l., Orta di Atella, CE, Italy) with reagents from Spinreact (Girona, Spain).

### Urine Analysis

Urinary samples were collected by cystocentesis for the obese group at T0 and T1 and for the CTR group only at the enrollment time (T0). Urinary protein/creatinine ratio (UP/C) was calculated starting from their spectrophotometric determination (OLOT, Spinreact) within 30 min.

### Insulin and Leptin Assay

Serum insulin analysis was performed for each sample using an ELISA kit (canine insulin ELISA, Mercodia AB). The insulin/glucose ratio (I/G) was calculated as the serum insulin (μU/ml) × 100/serum glucose (mg/dl), according to Bailhache et al. (51). In particular, the detection range was 2.3–173 mU/L with a detection limit of 1.15 mU/L. Absorbance was determined using a microplate reader (GDV programmable MPT Reader DV 990BV4, Agilent Technologies, Santa Clara, CA, USA) at 450 nm.

Serum leptin was estimated by an ELISA kit (Canine Leptin ELISA Cat. EZCL-31K, Millipore, Billerica, MA, USA). The detection limit was 0.2 ng/L, and intra- and interassay coefficients of variation (CV) were <5%. Absorbance was determined by a spectrophotometer with a wavelength of 450 nm (Epoch, BioTek Instruments Inc., Winooski, VT, USA).

### Inflammatory Cytokines Assay

Serum TNF-α and IL-6 were measured by canine cytokine ELISA kit (Genorise, Glen Mills, PA, USA). TNF-α detection range assay was 1–2,200 pg/ml with intra- and interassay CV <7 and <9%, respectively. IL-6 detection range assay was 50–3,200 pg/ml with intra- and interassay CV <6 and <9%, respectively. Absorbance was determined using a microplate reader (GDV programmable MPT Reader DV 990BV4, Agilent Technologies, Santa Clara, CA, USA) at 450 nm.

### Cardiovascular Biomarkers

Serum ET-1 was assessed using a specie-specific commercial sandwich ELISA kit (Claud-Clone Corp, Katy, TX, USA). The detection range was 6.17–500 pg/ml, and intra- and interassay CV were <10 and <12%, respectively. Absorbance was determined using a microplate reader (GDV programmable MPT Reader DV 990BV4, Agilent Technologies, Santa Clara, CA, USA) at 450 nm.

The serum troponin I levels were assessed using a human commercial kit [ADVIA Centaur High Sensitivity Troponin I (TNIH)], previously validated in canine species (52). The detection limit was 0.006 ng/ml. The analyses were performed using a chemiluminescence immunodiagnosis system (IMMULITE 1000 Systems, Siemens Healthcare S.r.l., Milan, Italy).

### Indirect Blood Pressure Measurement

The arterial blood pressure (ABP) was recorded for the obese group at T0 and T1 and for the CTR group only at the enrollment time (T0).

Blood pressure was measured by an automated oscillometric device (HDO, S + B MedVet). A cuff of appropriate size was placed on a limb or the tail, taking into account the dog body size. The first measurements were discarded, and a total of five to seven consecutive measurements were taken by the same operator, when the patient was calm after a short period of acclimatization in a quiet room, in ventral or lateral recumbency. The highest and lowest values of systolic, mean, and diastolic arterial blood pressure were ruled out, and an average value was obtained on the three remaining measurements. Dogs with systolic arterial blood pressure (SABP) >160 mmHg were considered to be affected by hypertension (53).

### Electrocardiography and Echocardiography

Electrocardiographic (ECG) and echocardiographic evaluations were performed for the obese group at T0 and T1 and for the CTR group only at the enrollment time (T0).

A standard six-lead electrocardiogram (ECG model 08SD, BTL Italia) was obtained with animals in right lateral recumbency, recording at least a 2-min strip (paper speed, 50 mm/s; calibration at 1 mV = 1 cm).

A conventional echocardiographic examination (two-dimensional, M-mode, spectral, and color flow Doppler) was performed with conscious dogs in lateral recumbency, using an ultrasound machine (Mylab50, Esaote) equipped with multifrequency (1–11 MHz) phased-array transducers. The echocardiographic study was performed in accordance with standardized methodologies (54–56) by the same operator (DP). For each measurement, the average value obtained from three consecutive cardiac cycles was used for analysis.

M-mode measurements included left ventricle free wall thickness at end diastole (LVFWd) and end systole (LVFWs), interventricular septal thickness at end diastole (IVSd) and end systole (IVSs), and LV internal diameter at end diastole (LVIDd) and at end systole (LVIDs). LVFWd and IVSd were indexed by allometric scaling based on body weight (57). The ratio of LV free wall thickness in diastole to LV internal diameter in diastole (LVFWd/LVIDd) and the ratio of interventricular septal thickness in diastole to LV internal diameter in diastole (IVSd/LVIDd) were estimated. The fractional shortening (FS), a parameter of LV systolic function, was obtained using the formula: [(LVIDd – LVIDs)/LVIDd] × 100. The Teicholz formula was used in order to calculate LV end-diastolic volume (EDV) and LV end-systolic volume (ESV) [EDV = (LVIDd^3^ × 7)/(LVIDd + 2.4); ESV = (LVIDs^3^ × 7)/(LVIDs + 2.4)]. End-diastolic volume index (EDVI) (normal value, <100 ml/m^2^) and end-systolic volume index (ESVI) (normal value, <30 ml/m^2^) were estimated dividing LV volumes by body surface area.

Two-dimensional measurements included left atrium/aorta diameter ratio (LA/AO) and ejection fraction (EF), calculated starting from the ventricular volumes obtained using the long-axis area-length (A-L) method.

Regarding spectral Doppler parameters, aortic and pulmonary peak velocities were measured from a left apical five-chamber view and a right short-axis view, respectively. Transmitral flow velocities were obtained using a left apical four-chamber view. Regarding the transmitral flow profile, the ratio of E wave peak velocity to A wave peak velocity (E/A) was obtained as a parameter of diastolic function; values were not used for analysis if the E and A waves were combined. Deceleration time (DT) was obtained from the E wave flow profile, considering the slope of the line starting from the maximal velocity E wave point to the baseline (56, 58).

In the obese group, indexed two-dimensional and mono-dimensional parameters, FS, FE (A-L), and spectral flow Doppler measurements were compared with those of the CTR group, and with the reference ranges, considering the actual weight of the dogs (57).

### Monoclonal Antibodies, Immunofluorescence, and Flow Cytometry

Peripheral blood CD21^+^ B cells, CD3^+^, CD3^+^CD4^+^, CD3^+^CD8^+^ T cells, CD4/CD8 ratio, and CD4^+^CD25^high^ Foxp3^+^Treg cells were evaluated by cytometry analysis (ATTUNE NxT FACS and Attune NxT Software, Life Technologies, Italy). Dead cells were ruled out as described (59, 60).

Fluorescein isothiocyanate (FITC), phycoerythrin (PE), and PE-cyanine7 anticanine CD3 (CA17.2A12 and CD3-12), CD4 (YKIX302.9), CD8 (YCATE55.9), and CD21 (CA2.1D6) monoclonal antibodies (mAbs) and isotype-matched mAb controls were used (Serotec Ltd London, UK). Foxp3 detection was measured using murine Foxp3 antibody (Clone FJK-16 s, eBioscience, San Diego, CA, USA), and the Foxp3detection Kit (Staining Set, eBioscience). Treg detection was performed as described (59, 61, 62).

### Statistical Analysis

Statistical evaluation was performed by Mann–Whitney test or Wilcoxon's matched pairs signed-rank test, as indicated. InStat version 3.0 software (GraphPad Software Inc., San Diego, CA, USA) was used. Results were considered significant at *p* < 0.05.

## Results and Discussion

### The Effect of a Weight Loss Program on Clinical Status of Enrolled Dogs

All enrolled dogs (obese and CTR group) were considered healthy based on clinical examination performed at the beginning of the weight loss period (T0). A second clinical examination at the end of the weight loss follow-up (T1) was made to determine the health state of obese dogs and to know the owner's impressions of the effects related to the weight loss of his pet. All obese dogs showed clinical improvement after the WLP. The mean of BCS recorded in the obese group at T0 was 8.18 (range, 7–9). At the end of the WLP (T1), all the dogs, with the exception of one that remained BCS 7, showed a reduction in BCS. In particular, the mean of BCS was 6.8 (BCS ranged between 5 and 8); 63.6% of the dogs showed a reduction of 1 point of BCS, 18.2% a decrease of 2 points, and 9.1% showed a decrease of 3 points. Besides, all owners emphasized many beneficial aspects related to the weight loss of their dogs. After 6 months of dietary treatment, 10 of the 11 owners reported that their dogs were more active, and the quality of their life was notably improved. The haircoat was shinier and softer in 7 of the 11 obese dogs. Other health benefits included a reduction in tachypnea (nine dogs) and breathing difficulties (one dog), and improvement of joint and locomotor problems (two dogs). It has already been observed that obese animals losing weight become more active and sociable (63). Excessive body weight is considered to be a risk factor for the development of osteoarthritis in men and dogs, and this pathological condition improves in obese dogs after weight loss (12). In our study, many adverse effects related to overweight status disappeared after weight loss, as already observed in the veterinary literature (64).

**Figure 1:**
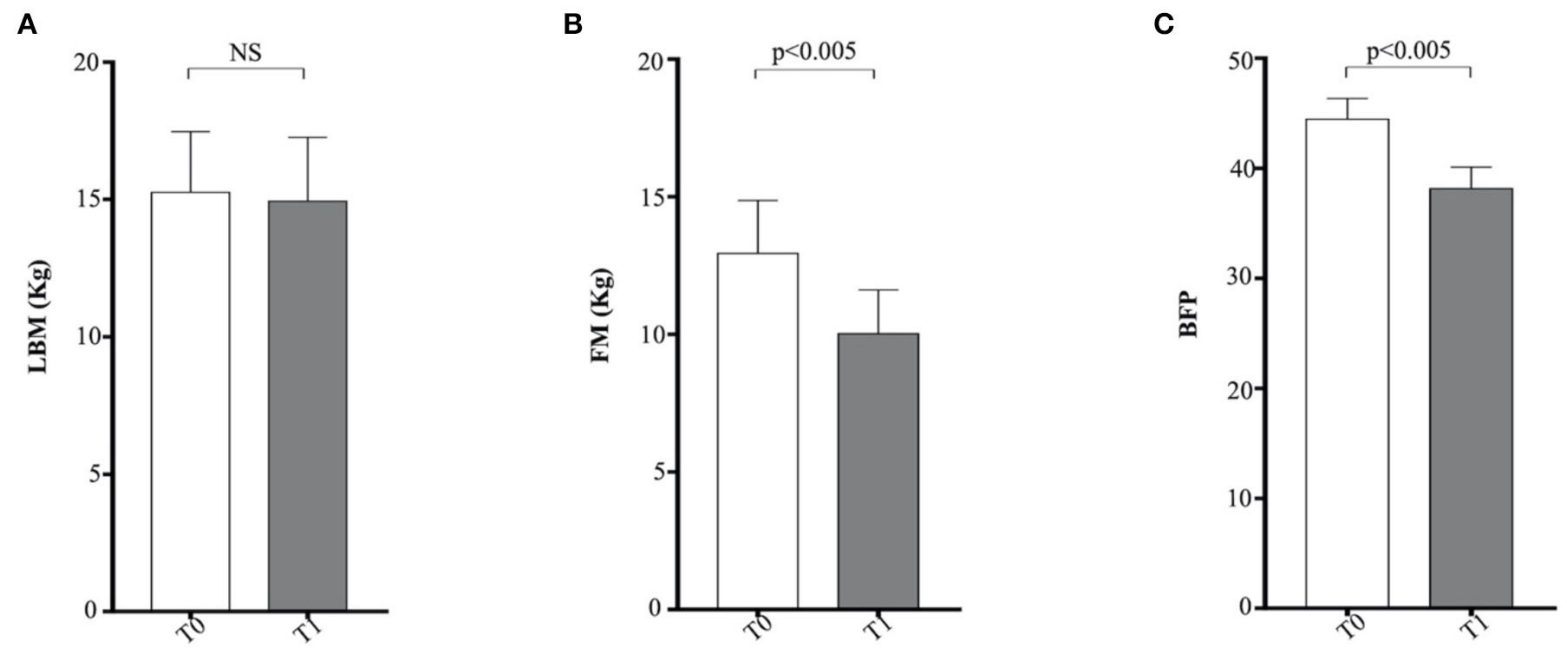
Morphometric equations in enrolled dogs. **(A)** LBM, lean body mass; **(B)** FM, fat mass; **(C)** BFP, body fat percentage. Obese dogs at T0, white column; obese dogs at T1, gray column. Statistics: Wilcoxon matched-pairs signed-rank test was used for T0 and T1 comparation in obese dogs. NS means not significant difference.

Finally, the FM values, expressed in kilograms, and BF, expressed as a percentage, obtained using equations developed by Witzel et al. (50) were significantly reduced at the end of the WLP (*p* < 0.005), but no significant correlation was found between leptin and LBM (*r* = −0.27, *P* = 0.167), FM (*r* = −0.16, *P* = 0.479), and BF (*r* = 0.14, *P* = 0.346), respectively (Figure 1). At present, although these new morphometric equations represent a quantitative and therefore more precise approach, they still cannot replace the subjective semiquantitative BCS system in the routine practice.

### Biochemical Profile of Enrolled Dogs Before and After Weight Loss Program

**Figure 2:**
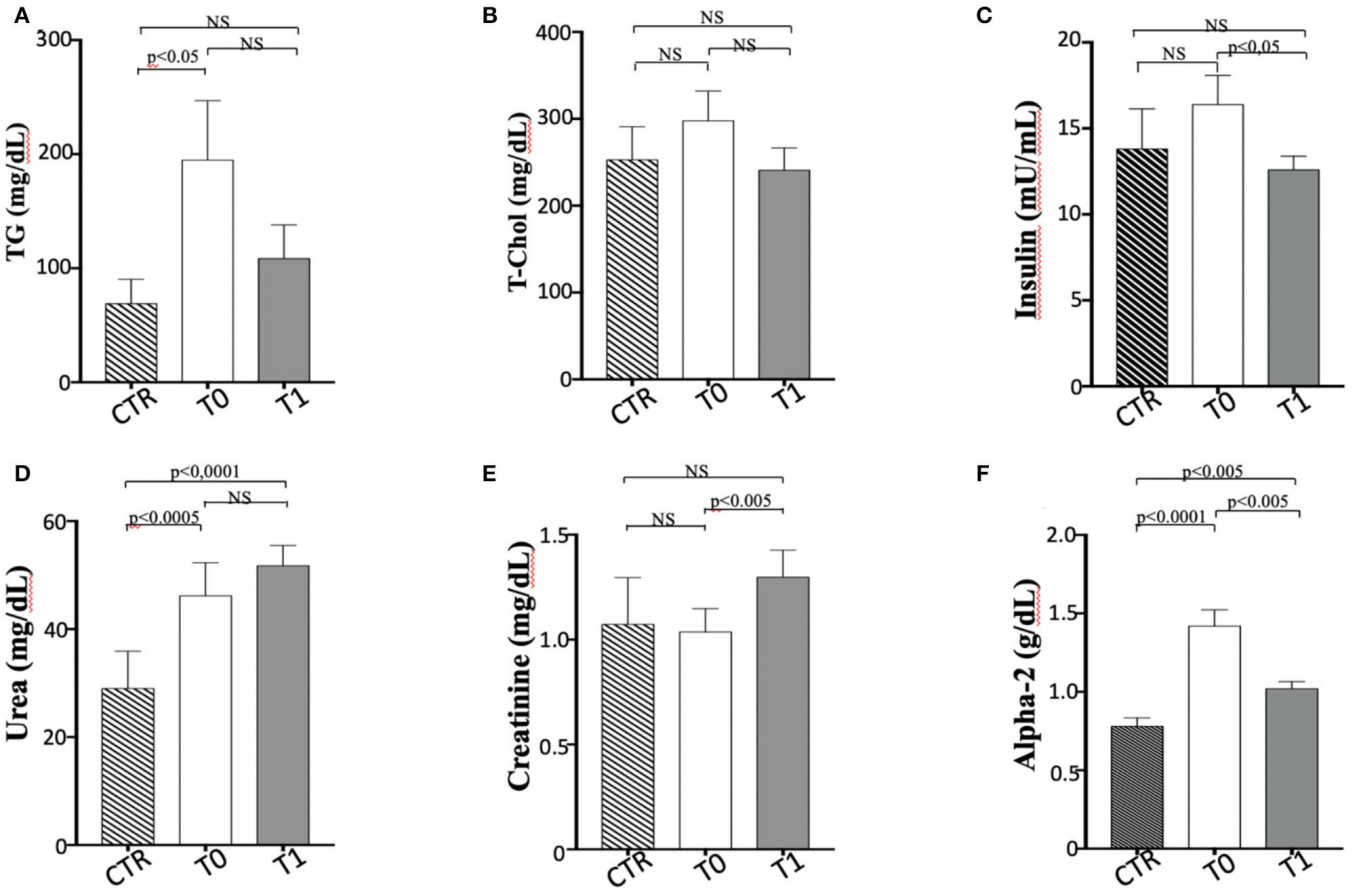
Biochemical parameters in enrolled dogs. **(A)** TG, Triglycerides; **(B)** T-Chol, total cholesterol; **(C)** Insulin; **(D)** Urea; **(E)** Creatinine; **(F)** Alpha 2-globulins. Control (CTR) group, dashed column; obese dogs at T0, white column; obese dogs at T1, gray column. Statistics: Wilcoxon matched-pairs signed-rank test was used for T0 and T1 comparation in obese dogs, while Mann-Whitney test was performed for the comparation between T0 obese dogs and CTR subjects. NS means not significant difference.

In this study, the metabolic panel (Table 2) confirmed that hyperlipidemia is a main serum biochemistry finding in obese dogs (65, 66). Serum triglycerides were significantly higher (*p* < 0.05) in the obese group at T0, if compared with the CTR group (Figure 2A). Dietary treatment improved metabolic status of OB dogs. WLP caused a non-significant decrease in triglycerides and cholesterol serum levels but a significant decrease (*p* < 0.05) in serum insulin in the obese dogs (Figures 2A–C). Only four dogs were categorized at T0 as being affected by ORMD. In association with the increased BCS, obese dogs showed high values for glucose (three dogs), total cholesterol (two dogs), triglycerides (four dogs), and SABP (one dog), and after the WLP, only two subjects met the ORMD classification criteria. It is noteworthy that it is still under discussion if this dysmetabolic status in dogs represents a set of risk factors as MetS in humans. This is because few studies addressed the frequency of comorbidities over the years and the effect of the increased body weight on life span (67). Potential renal damage could not be ruled out in obese dogs; microalbuminuria has been reported in obese human patients with MetS, suggesting an early onset of renal failure (68). In our obese cohort, urea was significantly higher at the two observation times (T0 *p* < 0.0005, T1 *p* < 0.0001) with respect to the CTR group, and serum creatinine significantly increased (*p* < 0.005) in the obese group after weight loss, even remaining in the reference range (Figures 2D,E).

**Table 2:** Biochemical profile in the control (CTR) group and obese group before and after the weight loss program (WLP).

**Parameter**	**Unit**	**CTR group**	**Obese group T0**	**Obese group T1**
Glucose	mg/dl	95.09 ± 8.2	96.57 ± 9.1	91.91 ± 11.1
Insulin	mU/ml	13.80 ± 2.3^ab^	16.38 ± 5.6^a^	12.58 ± 2.6^b^
I:G ratio	–	14.54 ± 2.8	17.08 ± 6.3	14.72 ± 5.3
Urea	mg/dl	29.00 ± 6.9^b^	46.16 ± 20.3^a^	51.73 ± 12.5^a^
Creatinine	mg/dl	1.07 ± 0.22^ab^	1.03 ± 0.37^a^	1.26 ± 0.39^b^
T-Chol	mg/dl	252.54 ± 38.2	297.73 ± 114	240.81 ± 85.5
TG	mg/dl	68.91 ± 21.4^b^	194.8 ± 172.9^a^	108.54 ± 97.7^ab^
ALT	UI/L	38.72 ± 7.2	56.4 ± 26.5	57.91 ± 20.83
ALP	UI/L	93.45 ± 47.6	264.9 ± 364.6	216.27 ± 303.0
T-Bil	mg/dl	0.313 ± 0.11	0.286 ± 0.19	0.237 ± 0.19
Na	mmol/L	146.36 ± 3.10	144.00 ± 2.86	146.18 ± 2.32
K	mmol/L	4.33 ± 0.35	4.27 ± 0.30	4.07 ± 0.24
TP	g/dl	6.63 ± 0.42	7.00 ± 0.55	6.24 ± 0.26
Alb	g/dl	3.44 ± 0.26	3.183 ± 0.52	3.097 ± 0.18
α1-glob	g/dl	0.297 ± 0.10	0.307 ± 0.13	0.230 ± 0.06
α2-glob	g/dl	0.780 ± 0.18^c^	1.418 ± 0.34^a^	1.022 ± 0.15^b^
β1-glob	g/dl	0.801 ±± 0.11	0.775 ± 0.11	0.632 ± 0.19
β2-glob	g/dl	0.689 ± 0.05	0.827 ± 0.23	0.723 ± 0.10
γ-glob	g/dl	0.600 ± 0.09	0.496 ± 0.17	0.512 ± 0.08

*Data are expressed as mean ± standard mean error. Along the row, different letters are used to indicate significant differences between groups at p < 0.05*.

*I/G ratio, insuline/glucose ratio; T-Chol, total cholesterol; TG, total triglycerides; ALT, alanine aminotransferase; ALP, alkaline phosphate; T-Bil, total bilirubin; TP, total proteins; Alb, albumins; α1-glob, α1-globulins; α2-glob, α2-globulins; β1-glob, β1-globulins; β2-glob, β2-globulins; γ-glob, γ-globulins*.

The elevated serum α 2-globulin fraction detected in the obese group at T0 could be related to the increased blood concentrations of acute phase proteins, suggestive of an inflammatory condition related to obesity condition (Figure 2F). It is particularly interesting that serum α 2-globulin fraction decreased significantly (*p* < 0.05) after weight loss (T1) in obese dogs, suggesting an anti-inflammatory action associated to dietary treatment (Figure 2F). Differences in the other biochemical parameters as well as in UP/C values (data not shown) between the two groups (obese and CTR) and in the obese group between the two observation times were not evident.

### The Effect of a Weight Loss Program on Cardiovascular Parameters of Enrolled Dogs

#### Cardiovascular Biomarkers

**Figure 3:**
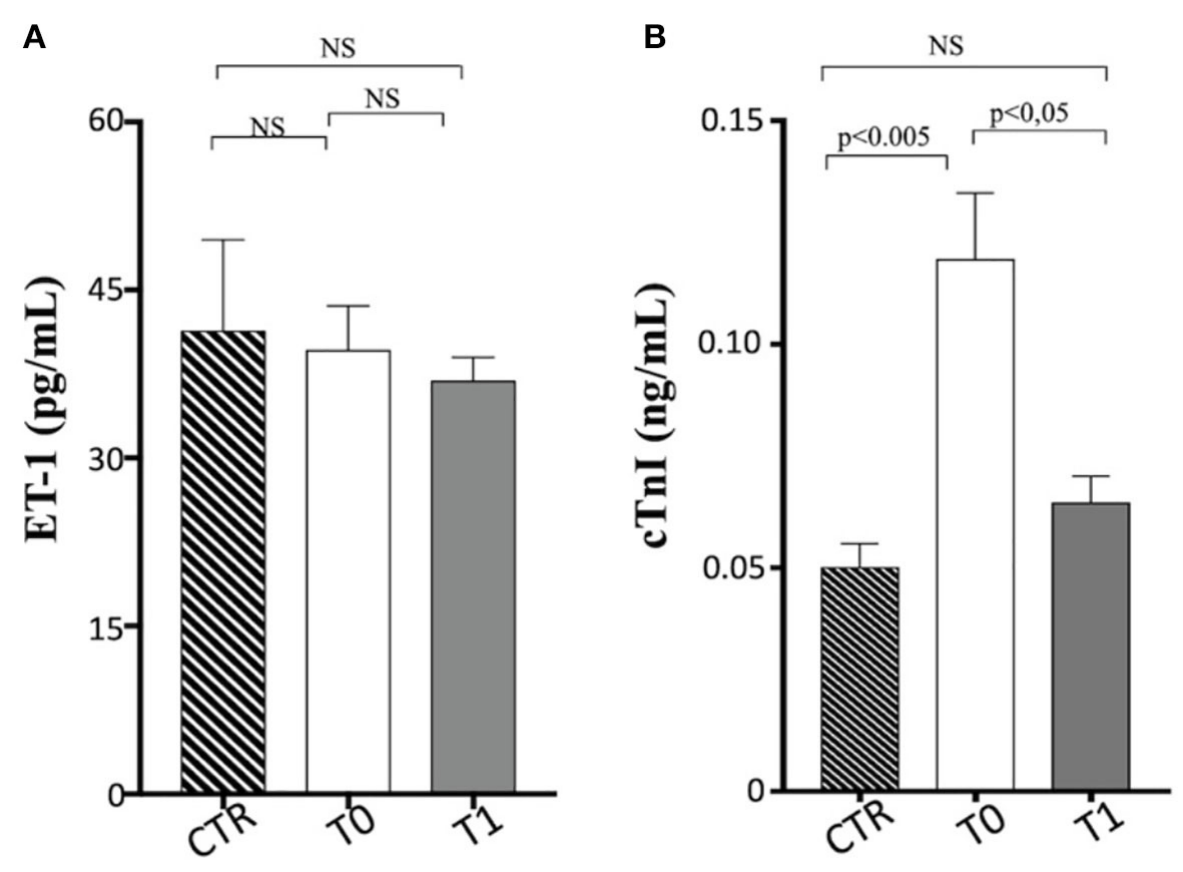
Cardiovascular biomarkers in enrolled dogs. **(A)** ET-1, endothelin-1; **(B)** cTnI, cardiac troponin I. Control (CTR) group, dashed column; obese dogs at T0, white column; obese dogs at T1, gray column. Statistics: Wilcoxon matched-pairs signed-rank test was used for T0 and T1 comparation in obese dogs, while Mann–Whitney test was performed for the comparation between T0 obese dogs and CTR subjects. NS means not significant difference.

Serum troponin levels were significantly greater in the obese dogs than in the CTR group, reducing with the weight loss (obese group at T0 vs. CTR group, *p* < 0.005; obese group at T0 vs. obese group at T1, *p* < 0.05), while ET-1 levels did not show differences between the groups (Table 3 and Figure 3). Cardiac troponin T (cTnT) and troponin I (cTnI) are globular myocardial proteins that are responsible for the control of the calcium binding between actin and myosin regulating the striated muscle contraction. Cardiac troponin I has not been found outside the heart and is a hallmark of myocardial injury in humans and small animals (69). In mice, experimental evidence proved that a high-fat diet lead to a myocardial damage mediated by the signal transducer (STAT)-3, a member of the STAT protein family, which can be activated by leptin and proinflammatory cytokines (70). In patients with acute inflammatory diseases, increased cTnI levels were correlated to the blood concentrations of proinflammatory cytokines (71). Lyngbakken et al. (72) described that obese humans experiment high-sensitivity cTnI levels higher than the normal threshold value, with a marked reduction following gastric bypass surgery in comparison to nutritional and lifestyle changes. In the veterinary literature, there is a unique study that investigated this biomarker in canine obesity. Cihan and Tural (73) found increased cTnI values in overweight animals than the normal weight counterpart, with positive correlation between cTnI and triglycerides levels. In our obese dogs, the baseline elevated cTnI levels appeared reduced after the WLP. These results may suggest that subclinical myocardial injury occurs in canine obesity, with recovering consequent to weight loss obtained by nutritional approach, although they must be confirmed by studies performed on a larger cohort of animals.

**Table 3:** Cardiovascular biomarkers in the control (CTR) and obese groups before and after the weight loss program (WLP).

**Parameter**	**Unit**	**CTR group**	**Obese group T0**	**Obese group T1**
ET-1	pg/ml	41.30 ± 8.17	39.58 ± 13.27	36.84 ± 7.06
cTnI	ng/ml	0.050 ± 0.017^b^	0.118 ± 0.044^a^	0.064 ± 0.018^b^

*Data are expressed as mean ± standard mean error. Along the row, different letters are used to indicate significant differences between groups at p < 0.05*.

*ET-1, endothelin-1; cTnI, cardiac troponin I*.

The occurrence of endothelial dysfunction is well-known in human's obesity and is related to an increased cardiovascular risk (74). Several experimental studies *in vivo* on human circulation have elucidated the complex mechanisms underlying these pathological aspects of the overweight status. The increased ET-1-dependent vasoconstriction is an important factor in the development of abnormal endothelial function and vasculopathies (coronary artery disease) observed in the obesity status. The enhancement of vasoconstrictor tone due to the increased activity of the ET-1 system has been reported in patients with “metabolically healthy obesity,” independently of arterial hypertension and other cardiovascular risk factors (75). The endothelial dysfunction is strictly related also to insulin resistance occurring in the MetS because an unbalance between ET-1 and nitric oxide endothelial production can lead to an impaired insulin-mediated vasodilation, reducing the peripheral glucose uptake (76). Furthermore, Carratù et al. (77) showed, in obese patients, high ET-1 levels associated with high systolic pulmonary artery pressure and obstructive sleep apnea. In dogs, some studies have investigated the ET-1 potential use for the diagnosis and prognosis of heart failure and other pathologies (such as pulmonary fibrosis and chronic hepatitis), but currently, it is not used in diagnostic routines (78–81). The ET-1 is able to distinguish between dogs with cardiac and non-cardiac dyspnea, but with lower sensitivity and specificity than other cardiovascular biomarkers (82). To the best of our knowledge, there is lack of data on ET-1 blood levels in natural canine obesity. In our population, ET-1 blood values did not appear significantly different in obese dogs after the WLP and compared to the CTR dogs. Indeed, only one examined obese dog had systemic hypertension at baseline, and none of them showed pulmonary artery hypertension assessed with echocardiography.

#### Electrocardiography and Echocardiography

The descriptive statistics of the examined cardiovascular parameters are shown in Table 4.

**Table 4:** Systolic blood pressure, heart rate, and echocardiographic findings in the control (CTR) and obese groups before and after the weight loss program (WLP).

**Parameter**	**Unit**	**CTR group**	**Obese group T0**	**Obese group T1**
SABP	mmHg	139.35 ± 15.09	145.64 ± 9.44	141.18 ± 14.27
HR	bpm	118.54 ± 15.89	128.64 ± 22.81	130.45 ± 15.88
LA/AO	–	1.44 ± 0.104	1.45 ± 0.119	1.44 ± 0.12
IVSd indexed	–	0.423 ± 0.06^b^	0.493 ± 0.06^a^	0.450 ± 0.08^a^
LVFWd indexed	–	0.452 ± 0.04	0.485 ± 0.06	0.472 ± 0.06
IVSd/LVIDd	–	0.254 ± 0.04^b^	0.334 ± 0.05^a^	0.282 ± 0.049^b^
LVFWd/LVIDd	–	0.263 ± 0.027^b^	0.321 ± 0.05^a^	0.287 ± 0.028 ^ab^
EDVI	ml/m^2^	54.53 ± 28.86	50.29 ± 28.49	50.63 ± 21.85
ESVI	ml/m^2^	21.74 ± 13.31^a^	14.72 ± 9.39^b^	16.33 ± 9.54^a^
FS	%	34.75 ± 5.64^b^	41.27 ± 7.85^a^	38.34 ± 5.74^ab^
EF (A-L)	%	64.84 ± 8.04^b^	72.09 ± 10.48^a^	69.48 ± 7.16^ab^
AO-PV	m/s	1.10 ± 0.218^b^	1.41 ± 0.29^a^	1.30 ± 0.132^a^
PO-PV	m/s	0.912 ± 0.190	0.994 ± 0.105	0.939 ± 0.103
E wave	m/s	0.720 ± 0.09	0.793 ± 0.167	0.778 ± 0.157
A wave	m/s	0.511 ± 0.102^b^	0.714 ± 0.124^a^	0.696 ± 0.110^a^
E/A	–	1.44 ± 0.227^a^	1.15 ± 0.37^b^	1.14 ± 0.301^b^
DT	ms	111.65 ± 13.33	120.93 ± 21.88	111.67 ± 7.17

*Data are expressed as mean ± standard mean error. Along the row, different letters are used to indicate significant differences between groups at p < 0.05*.

*SABP, systolic arterial blood pressure; HR, heart rate; LA/AO, left atrium to aorta diameter ratio; IVSd, interventricular septum at end-diastole; LVIFWd, left ventricle free wall at end-diastole; EDVI, end-diastolic volume index; ESVI, end-systolic volume index; FS, fractional shortening; EF (A-L), ejection fraction calculated with area-length method; AO-PV, aortic peak velocity; PO-PV, pulmonary peak velocity; E wave, E wave peak velocity; A wave, A wave peak velocity; E/A, E wave peak velocity to A wave peak velocity ratio; DT, deceleration time*.

In the obese group at T0, ECG revealed five animals with no respiratory sinus arrhythmia, two dogs with sinus tachycardia, one dog with features of left ventricular enlargement, two dogs with low R wave amplitude, and four dogs with features of myocardial microinfarcts. No ECG abnormalities were recorded in the CTR group. The mean electrical axis was in the normal range in both groups, and the heart rate was not significantly different between groups.

SABP values were not significantly different between groups. Only one obese dog was hypertensive (164 mmHg) at T0 and showed SABP values just below the cutoff limit (158 mmHg) after the WLP.

**Figure 4:**
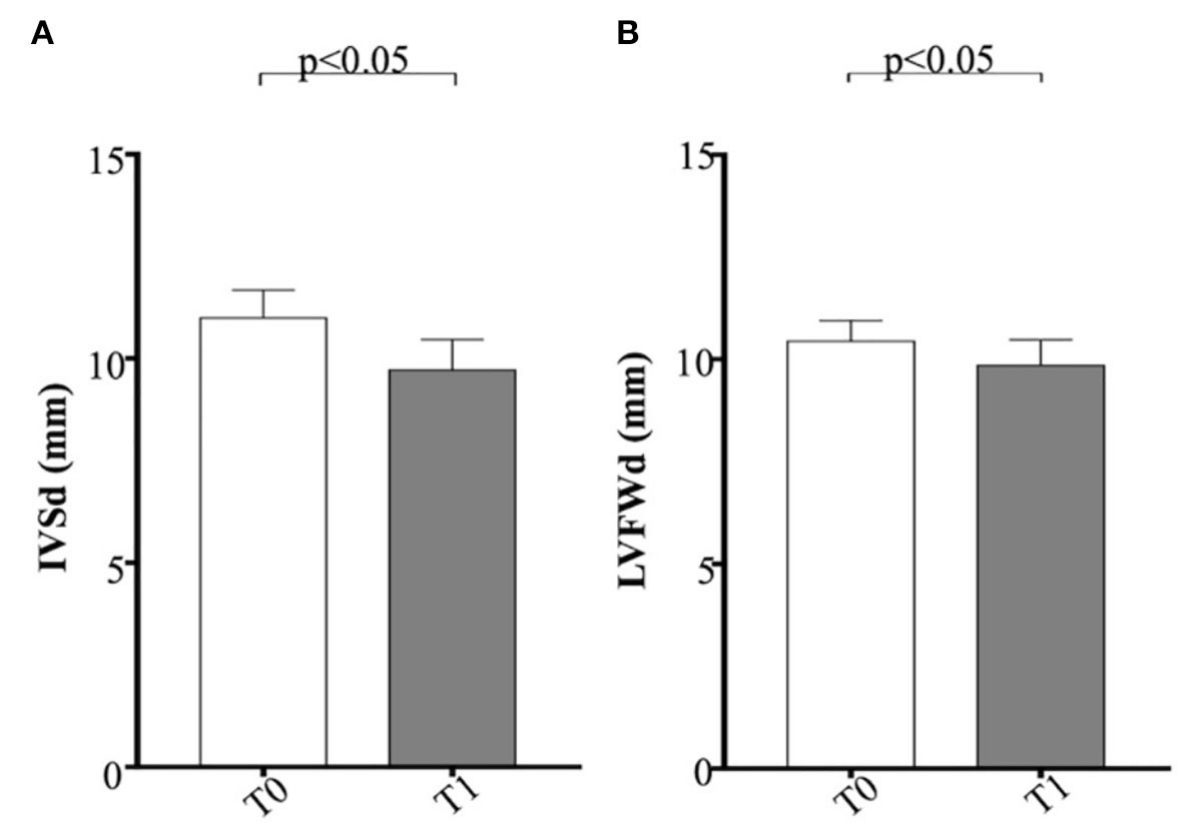
Echocardiographic parameters in enrolled dogs. **(A)** IVSd, interventricular septum at end diastole; **(B)** LVFWd, left ventricle free wall at end diastole. Obese dogs at T0, white column; obese dogs at T1, gray column. Statistics: Wilcoxon matched-pairs signed-rank test was used for T0 and T1 comparation in obese dogs. NS means not significant difference.

**Figure 5:**
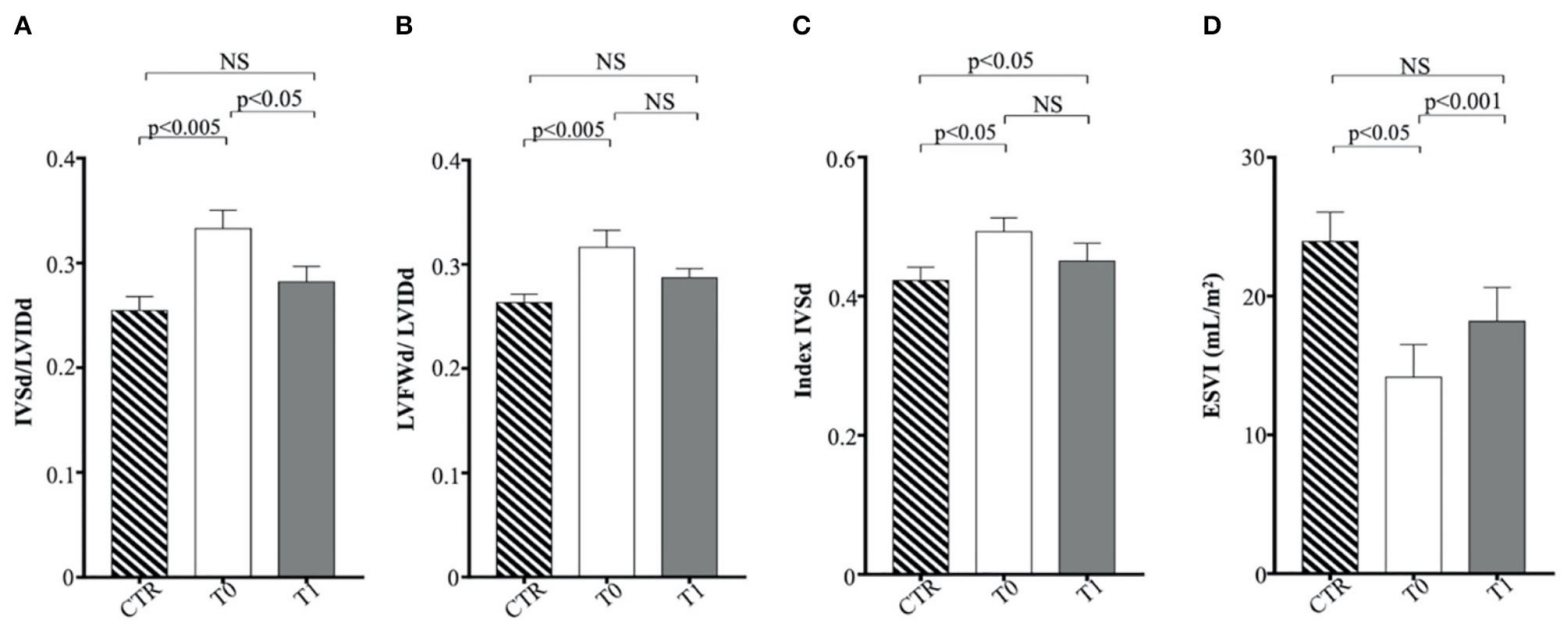
Echocardiographic parameters in enrolled dogs. **(A)** IVSd/LVIDd, ratio of interventricular septal thickness to left ventricular internal dimension at end diastole; **(B)** LFVFWd/LVIDd, ratio of left ventricular free wall thickness to left ventricular internal dimension at end diastole; **(C)** indexed IVSd, indexed interventricular septum thickness at end diastole; **(D)** ESVI, end-systolic volume index. Control (CTR) group, dashed column; obese dogs at T0, white column; obese dogs at T1, gray column. Statistics: Wilcoxon matched-pairs signed-rank test was used for T0 and T1 comparation in obese dogs, while Mann–Whitney test was performed for the comparation between T0 obese dogs and CTR subjects. NS means not significant difference.

Regarding M-mode and 2D echocardiographic measurements, IVSd and LVFWd decreased in the obese group after the WLP (*p* < 0.05) (Figures 4A,B). The IVSd/LVIDd ratio was significantly greater in the obese group at T0 than CTR group (*p* < 0.005) and decreased in obese dogs after the WLP (*p* < 0.05); the LVFWd/LVIDd ratio was significantly greater in the obese group at T0 than in the CTR group (*p* < 0.005) and was reduced after weight loss at values comparable with the normal weight dogs but without statistical significance (Figures 5A,B). The indexed IVSd was greater in the obese subjects at T0 (*p* < 0.05) and T1 (*p* < 0.05) than in the CTR group (Figure 5C); four obese dogs showed at T0 the indexed IVSd higher than the cutoff value (0.52), which returned below the normal limit only in one subject after the WLP. No statistical differences were observed for indexed LVFWd; two obese dogs showed at T0 the indexed LVFWd higher than the threshold value (0.53), which was not reduced below the normal limit after the WLP.

**Figure 6:**
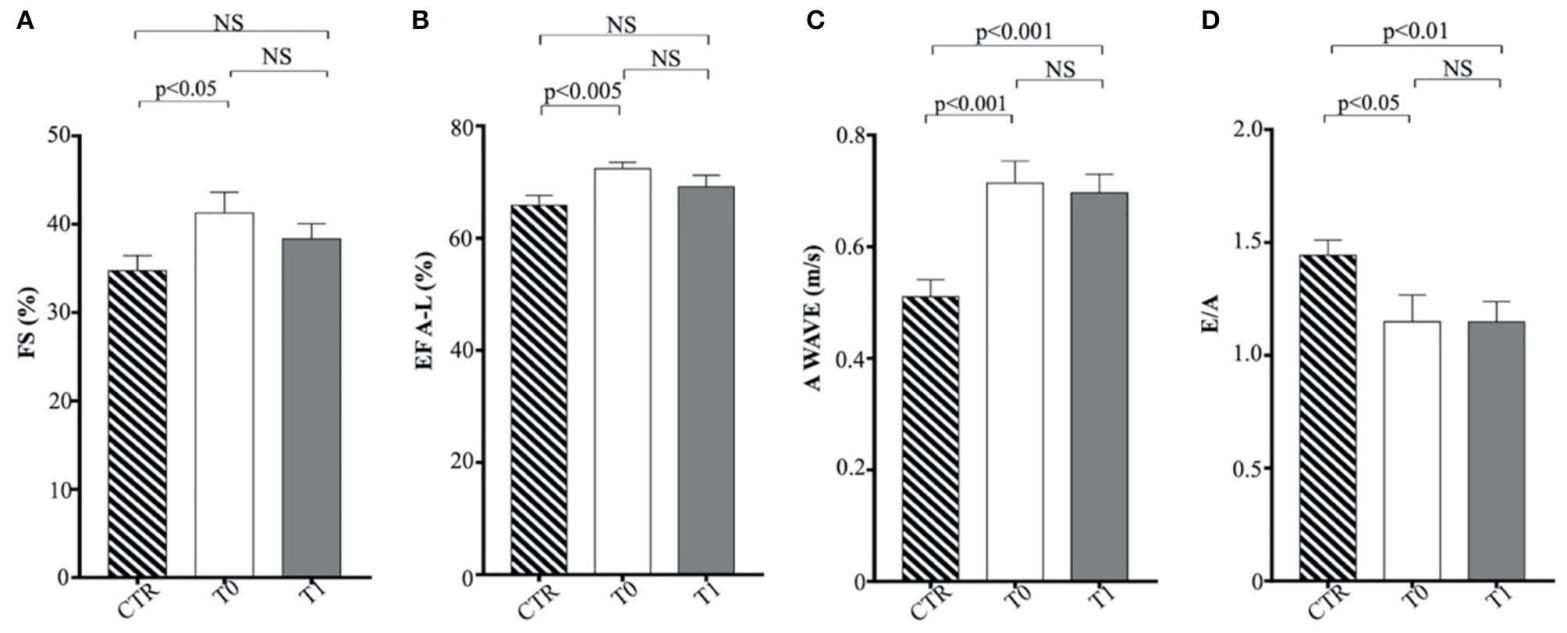
Echocardiographic parameters in enrolled dogs. **(A)** FS, fractional shortening; **(B)** EF (A–L), ejection fraction calculated with area-length method; **(C)** A wave, A wave peak velocity; **(D)** E/A, ratio of peak early to peak late left ventricular inflow velocities. Control (CTR) group, dashed column; obese dogs at T0, white column; obese dogs at T1, gray column. Statistics: Wilcoxon matched-pairs signed-rank test was used for T0 and T1 comparation in obese dogs, while Mann–Whitney test was performed for the comparation between T0 obese dogs and CTR subjects. NS means not significant difference.

The indexed LV end-systolic volume (ESVI) was greater in the CTR group compared to obese animals at T0 (*p* < 0.05) and significantly increased after the WLP (*p* < 0.001) (Figure 5D). There was no statistical differences for EDVI. The values of EDVI and ESVI were not greater than the normal threshold value in any animal belonging to the different studied groups. Obese dogs at T0 showed significantly higher FS percentage (*p* < 0.05) and EF (*p* < 0.005) percentage measured by the long-axis area-length method than the CTR dogs, the percentages of which decreased after WLP but without statistical significance (Figures 6A,B).

Regarding Doppler echocardiography, AO-PV was higher in the obese group at T0 (*p* < 0.01) and T1 (*p* < 0.01) in comparison to the control animals. The obese dogs had significantly lower E/A ratio at T0 (*p* < 0.05) and T1 (*p* < 0.01) than the CTR group (Figures 6C,D). No statistical significance was observed for DT and the remaining echocardiographic variables.

A high proportion of obese people develops, over the years, cardiovascular pathologies (hypertension, coronary artery disease, atrial fibrillation, heart failure) with a shortened lifespan (83). Obesity represents a stressing factor for the cardiovascular systems with negative effects on cardiac morphology and function (24). The complex peripheral and central hemodynamic alterations due to increased fat mass lead to a higher LV stroke volume with consequent change in LV geometry and the onset of systolic and diastolic dysfunction. The cardiac remodeling can occur as eccentric or concentric LV hypertrophy, with the prevalence of this latter pattern (84). It must be underlined that cardiac remodeling may occur in overweight patients also regardless of obesity-related comorbidities. By evaluating cardiac function with standard echocardiography, obese humans do not show alterations of systolic parameters, which can be also increased. Advanced echocardiographic methodologies, such as tissue Doppler velocity and strain and speckle tracking, allow to detect subtle worsening of the systolic LV performance (85). In humans, surgical weight reduction is accompanied by a decrease in the LV eccentric hypertrophy and an improvement of the ejection fraction (86). In canine species, some authors showed the occurrence of some aspects of LV concentric hypertrophy and hypertension in a number of obese animals (19, 20, 23). In an experimental dietary program, Pelosi et al. (25) reported, in Beagle dogs, an increase in the LV myocardial cross-sectional area with weight gain, which reversed completely with weight loss in association with physical exercise. On the contrary, Adolphe et al. (87) described only a partial recovery of LV concentric hypertrophy after the weight loss phase in a short-term experimental obesity model. In our naturally obese dogs, IVSd an LVFWd decreased overall after WLP, but only in one animal the indexed IVSDd returned below the normal limit. According to other authors (20, 23), the obese dogs studied did not show eccentric hypertrophy compared to the CTR dogs at the two time points, showing an increasing in the ESVI value after WLP. Conventional parameters of systolic function, FS and EF, were increased in obese than the normal weight counterpart according to Tropf et al. (20), but no significative differences were observed after WLP. On the contrary, Broussard et al. (18) evidenced a worsening in the circumferential strain and LV torsion using magnetic resonance in dogs experimentally fed with a diet containing saturated and monounsaturated fatty acids. Regarding diastolic function, we observed a reduced E/A ratio than normal weight dogs previously described in dogs and humans (20, 84), without a significant improvement at the second time point. Only one animal in our population was hypertensive and did not show a marked reduction in values after WLP. The analysis of our results and the few data available in the literature suggest that hypertension and cardiac changes are present in canine obesity, but probably in a subset of subjects having the overweight condition a less heavy impact on the cardiovascular apparatus than humans. In our natural model of obesity, the WLP was accompanied by only a partial recovering of the LV remodeling, although a greater effect cannot be excluded in case of longer dietary treatment. Further investigations using advanced echocardiographic techniques need to assess the occurrence of subtle systolic dysfunction in naturally obese dogs.

#### The Immune Profile of Enrolled Dogs Upon WLP

**Figure 7:**
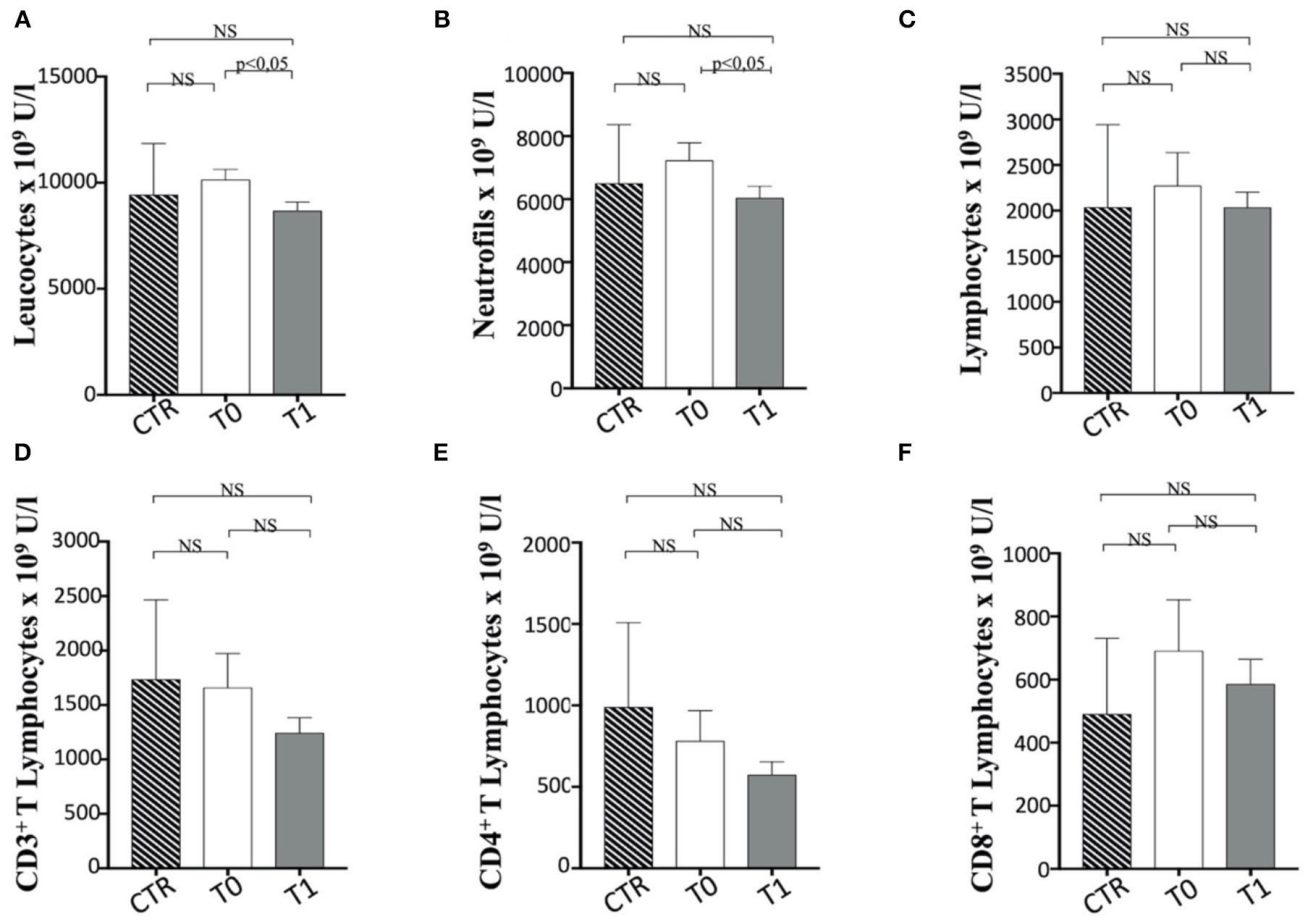
The number of Leucocytes, neutrophils and T cell subsets in enrolled dogs. The figure reports the evaluation on the number of **(A)** leukocytes, **(B)** neutrophils, **(C)** total lymphocytes, **(D)** CD3^+^, **(E)** CD4^+^, and **(F)** CD8^+^ T cell subsets in obese dogs, at T0 and T1, and control (CTR) subjects. Referred values in “*n* × 10^9^ U/L” indicate results obtained in CTR group (dashed column), in obese dogs at T0 (white column) and T1 (gray column), as indicated. Statistics: Wilcoxon matched pairs signed-rank test was used for T0 and T1 comparation in obese dogs, while Mann-Whitney test was performed for the comparation between T0 obese dogs and CTR subjects. NS means not significant difference.

We performed the first approach of immune profile evaluation of obese dogs subjected to WLP basing on the peripheral blood leukocyte count. In this perspective, it appeared that obese group at T0 (before the start of the WLP) showed a higher (not statistically significant) leukocyte number than healthy weight dogs (CTR group) (Figure 7A). Following the WLP (T1), enrolled dogs showed a statistically significant reduction in the total number of leukocytes compared with T0, at similar levels to those manifested in the CTR group (Figure 7A). In particular, neutrophils and not lymphocytes were reduced in absolute number in obese group dogs following the WLP (Figures 7B,C, respectively). This occurrence would suggest normalization of leukocyte levels following the WLP, which coincides with the clinically observed weight reduction in enrolled dogs. No change was observed in the platelet number in obese dogs, which, at T0, was at levels similar to those of the CTR dogs and was unmodified at T1 (data not shown).

In a second approach, the evaluation of the number of T lymphocyte subpopulations was considered. In this context, the total number of CD3^+^ T cells appeared to be unmodified during WLP. CD3^+^ CD4^+^ T and CD3^+^ CD8^+^ T cell numbers appear to be in a decreasing trend after WLP in the obese group, although not statistically significant (Figures 7D–F).

**Figure 8:**
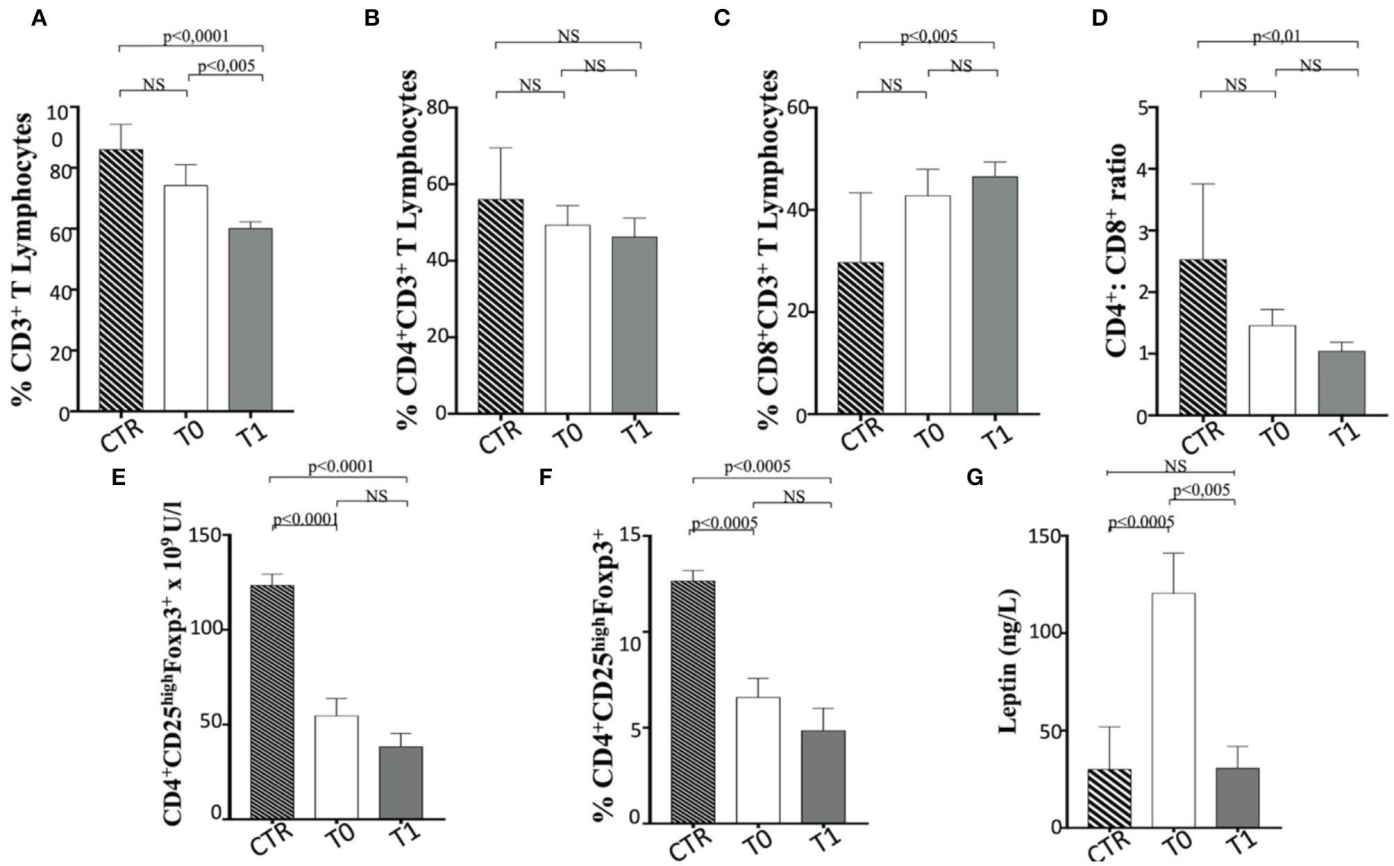
The asset of T cell subsets and the serum level of leptin in enrolled dogs. **(A–C)** Percentage of total CD3^+^, CD4^+^, and CD8^+^ T lymphocytes in obese dogs, at T0 and T1, and control (CTR) subjects. **(D)** CD4^+^ CD8^+^ ratio. **(E,F)** Number (as *n* × 10^9^ U/L) and percentage of Treg cells (indicated as CD4^+^ CD25^high^ FoxP3^+^ T cells), in obese, at T0 and T1, and CTR subjects. Referred values indicate results obtained in CTR group (dashed column), in obese dogs at T0 (white column) and T1 (gray column), as indicated. **(G)** Serum leptin level in obese dogs, at T0 (white column) and T1 (gray column), and CTR (dashed column) subjects. Statistics: Wilcoxon matched-pairs signed-rank test was used for T0 and T1 comparation in obese dogs, while Mann-Whitney test was performed for the comparation between T0 obese and CTR subjects. NS means not significant difference.

When the percentage of total CD3^+^ T lymphocytes is considered, a significant reduction between T0 and T1 was observed in dogs following the WLP. In contrast, subjects at T1 showed a lower CD3^+^ T cell percentage than the CTR dogs (Figure 8A). It is of note that only the CD3^+^ CD8^+^ and not CD3^+^ CD4^+^ T cells increased their percentage in obese dogs at T1 when compared with the CTR group subjects (Figures 8B,C), as also confirmed by the reduction in CD4^+^/CD8^+^ T cell ratio that decreased between T0 and T1, which could be due to CD8^+^ T cells (Figure 8D). It is worth noting that the obese group at T0 showed a higher percentage of CD3^+^ CD8^+^ T cells and a lower CD4^+^/CD8^+^ T cell ratio than the CTR group dogs, although not statistically significant (Figures 8B,D). This aspect could reflect a potential tendency of obese dogs to exhibit immune-mediated pathophysiologies (31, 67, 88, 89). This is probably related to an exacerbation of the cytotoxic and secretory functions of CD8^+^ T lymphocytes or, as in the case of dogs enrolled in this trial, a potential dysregulation of the immune response due to population increase in T CD8^+^. In this sense, the WLP would not have reached a recovery at 6 months (T1), but probably a longer period (at least 12–18 months) could have determined a homeostatic recovery.

Notably, the Treg cells (CD3^+^ CD4^+^ CD25^+^ FoxP3^+^) appeared reduced in number (Figure 8E) and percentage (Figure 8F) in obese dogs, when compared to healthy dogs (CTR group). Such evidence could indicate a tendency of overweight dogs to exhibit immune dysregulation and subsequent autoimmune phenomena (31, 90, 91). However, the WLP of obese dogs at 6 months (T1) failed in inducing the normalization of Treg cells, maintaining the numerical and percentage levels lower than CTR dogs (Figures 8E,F). It is of note that serum leptin level in obese subjects at T0 was higher than that in the CTR dogs, while it was reduced at T1 assuming values comparable to those of the CTR subjects (Table 4 and Figure 8G). In this context, an inverse leptin and Treg cell correlation (90, 91) is usually recorded in humans, although there is no definitive evidence on this phenomenon in dogs (37). It is conceivable that a longer period of WLP, with the persisting normal level of leptin, could also generate the Treg recover.

**Figure 9:**
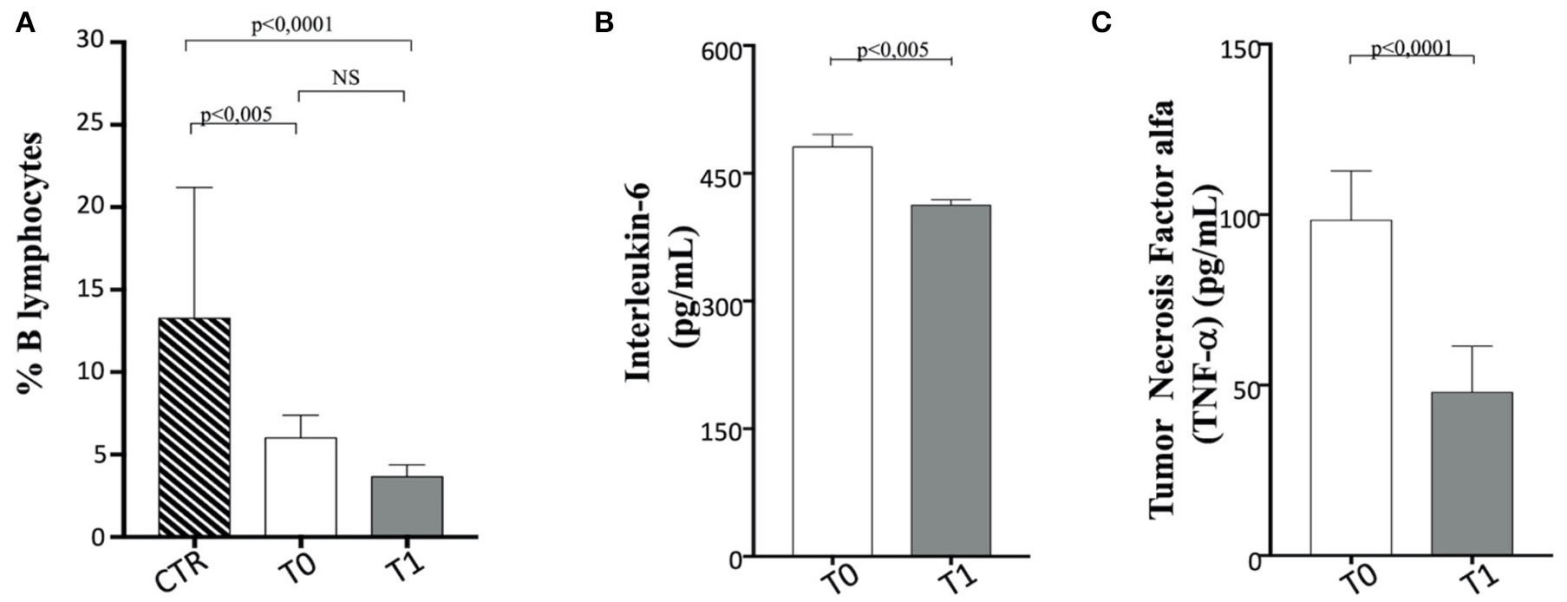
B cells, interleukin (IL)-6, and tumor necrosis factor (TNF)-α in enrolled dogs. **(A)** Evaluation of B cell percentage in obese dogs, at T0 (white column) and T1 (gray column) and control (CTR) (dashed column) subjects, as indicated. **(B,C)** Amplitude of IL-6 and TNF-a production, respectively, in obese dogs at T0 (white column) and T1 (gray column). Values are in pg/ml. Statistics: Wilcoxon matched-pairs signed-rank test was used for T0 and T1 comparation in obese dogs, while Mann–Whitney test was performed for the comparation between T0 obese and CTR group subjects. NS means not significant difference.

No variation in B cell percentage was found in the before and after WLP, although it appears reduced in the obese group compared to the CTR dogs (Figure 9A).

When serum proinflammatory cytokine (30) levels are assessed, it is worthy of interest that IL-6 and TNF-α appeared to decrease significantly following WLP (T0 vs. T1) in obese dogs (Figures 9B,C and Table 5). This evidence may reflect a desirable reduction in inflammatory immune dysregulation in obese dogs following WLP. Besides, the here observed cytokine decrease could be correlated to serum leptin reduction (92, 93).

**Table 5:** Leptin and inflammatory cytokines in the control (CTR) and obese groups before and after the weight loss program (WLP).

**Parameter**	**Unit**	**CTR group**	**Obese group T0**	**Obese group T1**
Leptin	ng/L	30.06 ± 5.64^b^	120.40 ± 20.64^a^	33.36 ± 12.04^b^
IL-6	pg/ml	–	480.83 ± 46.65^a^	412.60 ± 21.21^b^
TNF-α	pg/ml	–	98.30 ± 43.64^a^	34.93 ± 14.63^b^

*Data are expressed as mean ± standard mean error. Along the row, different letters are used to indicate significant differences between groups at p < 0.05. IL-6 and TNF-α levels were not measured in the CTR group*.

*IL-6, interleukin-6; TNF-α, tumor necrosis factor-α*.

## Limitations

One important limitation of this non-experimental study was the short term (6 months) of WLP, since the dogs were unfollowed until they achieved an ideal body condition. The average duration of a complete weight loss period, able to conduce dogs to a healthy weight, is 9 months, and some dogs need more than 12 months to reach the target (94). Therefore, enrolled obese dogs might have more benefits if the study had been prolonged, especially to restore their immunological balance. In addition, gender differences for the investigated parameters were not evaluated because the genders were not balanced in the two study groups.

Finally, the degree of weight loss achieved and, consequently, the immunological, biochemical, and cardiovascular findings observed may also be affected by timing and duration of obesity, which was not possible to ascertain accurately based on the information provided by the dog's owners.

## Conclusions

In conclusion, in the present study, weight loss was associated by an improvement in both activity and global life quality of dogs. Furthermore, the metabolic status of obese dogs, with particular regard to dyslipidemia, improved after WLP.

Regarding the cardiovascular system, obese dogs can show systolic hypertension and LV remodeling, although probably the overweight condition has a less impact than humans. However, cardiovascular assessment should not be omitted in the evaluation of the clinical status of obese dogs. In our natural model of obesity, we observed only a partial recovery of the LV remodeling, although a greater effect cannot be ruled out if the dietary program had been longer.

Regarding the immunological status of obese dogs, our observations indicate that WLP, obtained in 6 months with a calorie-restricted diet, can modify the immune structure and, in some aspects, induce an overall homeostatic recovery of the canine immune response. The total number of leukocytes in obese dogs subjected to WLP tends to be similar to that observed in normal-weight subjects. Furthermore, WLP reduced the serum presence of proinflammatory cytokines, at least for those observed in the present trial (IL-6 and TNF-α). However, the evidence of a percentage increase in CD8^+^ T lymphocytes is worthy of interest, and further studies are needed to understand the potential homeostatic significance. Besides, the WLP significantly reduced the serum concentration of leptin, which has been very high at the beginning of the WLP, and after 6 months of treatment, it became similar to those observed in normal-weight dogs. Therefore, calorie restriction appeared to induce potentially beneficial effects, since it promoted a homeostatic recovery of the structure of the immune response.

The WLP for only 6 months was unable to normalize the levels of Treg, but we cannot exclude that a more prolonged treatment could have obtained this desirable effect. However, the reduction in serum leptin could also have induced the observed decreased proinflammatory cytokine production, as previously described. In this context, it is of fundamental relevance to suggest to the scientific veterinary community an in-depth study to understand the relationship between leptin and Treg cells in the dog, where, in humans, this aspect is particularly relevant and universally recognized as the cause of disease when altered.

This study opens new perspectives in the understanding of the relationship between diet and immune response in dogs, where the reference scientific literature is not sufficiently able to explain the effects of calorie restriction in the treatment of obesity in relation to the morbidities and pathophysiological aspects frequently associated to canine weight gain.

## Data Availability Statement

The raw data supporting the conclusions of this article will be made available by the authors, without undue reservation.

## Ethics Statement

The animal study was reviewed and approved by Ethical Animal Care and Use Committee of the University of Naples Federico II. Written informed consent was obtained from the owners for the participation of their animals in this study.

## Author Contributions

LC, DP, GR, and GT contributed to the conception of the experimental design of the study. LC, DP, and PL participated in the clinical management of the dogs. DP performed the echocardiographic examinations. LC, NM, and PL performed the hematological and biochemical analysis, ELISA tests, and analyzed the data. AP, FC, VR, and AG performed the immunological research and analyzed the data. FF organized the database. AP and NM performed the statistical analysis. LC, DP, AP, GR, YA, PL, and GT wrote the manuscript. All authors contributed to the article and approved the submitted version.

## Conflict of Interest

The authors declare that the research was conducted in the absence of any commercial or financial relationships that could be construed as a potential conflict of interest.
